# A pilot study on molecular diagnosis of *Hapalotrema mistroides* (Digenea: Spirorchiidae) infection in blood samples of live loggerhead turtles *Caretta caretta*

**DOI:** 10.1186/s12917-020-2232-y

**Published:** 2020-01-14

**Authors:** Erica Marchiori, Giorgia Dotto, Cinzia Tessarin, Mario Santoro, Andrea Affuso, Luciano Tarricone, Ludovica Di Renzo, Daniela Freggi, Vincenzoleo Spoto, Federica Marcer

**Affiliations:** 1 Department of Animal Medicine, Production and Health, University of Padova, Viale dell’Università 16, 35020 Legnaro, PD Italy; 2 Department of Integrative Marine Ecology, Stazione Zoologica Anton Dohrn, Villa Comunale 1, 80121 Naples, Italy; 3 Centro Ricerche Tartarughe Marine, Stazione Zoologica Anton Dohrn, Via Nuova Macello 16, 80055 Portici, NA Italy; 4 Veterinary practitioner, Centro Recupero Il Benvenuto, S.S. 16 2287/C, 45038 Polesella, RO Italy; 5 Istituto Zooprofilattico Sperimentale Abruzzo e Molise “G. Caporale”, Via Campo Boario, 64100 Teramo, Italy; 6 Centro Recupero e Riabilitazione Tartarughe Marine “L. Cagnolaro” Centro Studi Cetacei Onlus, Via di Sotto, 65125 Pescara, Italy; 7 Centro Recupero Tartarughe Marine di Lampedusa, Lungomare L. Rizzo, 92010 Lampedusa, AG Italy

**Keywords:** Spirorchiidae, *Caretta caretta*, Real time PCR, Circulating DNA

## Abstract

**Background:**

Parasites of the family Spirorchiidae cause disease and mortality in marine and freshwater turtles; two species, *Hapalotrema mistroides* and *Neospirorchis* sp., are reported in the resident population of loggerhead turtles of the Mediterranean Sea, with the first being the most widespread. In vivo diagnosis of spirorchidiasis can represent a challenge in guaranteeing prompt control and treatment of the disease and is currently limited to copromicroscopy.

The aim of this study was the development of a real time PCR assay with TaqMan probe for the detection of *H. mistroides* infection in the blood of live loggerhead turtles, *Caretta caretta,* hospitalized in rehabilitation centres. Its potential use for in vivo diagnosis is explored.

**Results:**

The developed real time PCR successfully detected *H. mistroides* DNA from both positive controls and experimental blood samples of live loggerhead sea turtles, showing good specificity, sensitivity and good reaction efficiency. Two out of three turtles which had demonstrated positivity at copromicroscopy also tested positive to this blood assay; DNA of *H*. *mistroides* was detected within the blood of one sea turtle, which tested negative for copromicroscopy.

**Conclusions:**

This study describes a specific and rapid molecular assay to detect *H. mistroides* infection from live sea turtles and highlights for the first time the presence of DNA of this species in turtle blood samples. Since this assay is able to detect low amounts of the parasitic free DNA in blood samples, its application could be helpful for in vivo diagnosis of *H. mistroides* infection as well as for epidemiological purposes.

## Background

Flukes of the family Spirorchiidae infect the cardiovascular system of marine and freshwater turtles [1, 2]. Pathological changes have been widely described in marine turtles, arising from the localization of the adults in blood vessels and the embolization of the eggs in the organs. The granulomatous reaction linked to egg accumulation can produce grossly visible lesions, which can disrupt organ anatomy and function [3, 4]. Post mortem diagnosis is generally achieved by the observation of typical gross lesions during necropsy and by histopathology; new molecular tools have been recently developed, demonstrating higher sensitivity than traditional methods and offering at the same time the plus of a genus or species-specific detection of the parasites lodged in the organs [5]. However, in vivo diagnosis of spirorchiidosis represents a challenge for the correct management of rescued sea turtles. Coprological examination is commonly used for detecting the species whose eggs accumulate within gastrointestinal walls and are shed in the environment through feces. Nevertheless, frequency of emission of eggs has not been ascertained, leaving space for false negative results when examining single aliquots of feces, which in turn is not always easily obtainable [6]. Moreover, the morphological similarity of eggs belonging to different spirorchiid genera (i.e. *Amphiorchis, Hapalotrema*, *Learedius* and *Monticellius*) requires an extra molecular step to reach a specific diagnosis. Presence of anti-blood flukes immunoglobulins directed towards soluble worm and egg antigens has been also tested in live sea turtles through direct and indirect ELISA [7–9]. Major limits of this approach are the potential cross-reactivity with gastrointestinal helminths in co-infected turtles, and the lack of a species specific diagnosis.

The aim of this study was to develop a sensitive assay for the detection of spirorchiid infection in blood samples of live loggerhead turtles *Caretta caretta* from Italian rehabilitation centres, and its potential for in vivo diagnosis*.* Based on current knowledge of epidemiology of spirorchiidosis within the Mediterranean Sea [4, 10], this study has been focused on the detection of infections by *Hapalotrema mistroides* which is the most common blood fluke species reported in this basin.

## Results

**Fig. 1:**
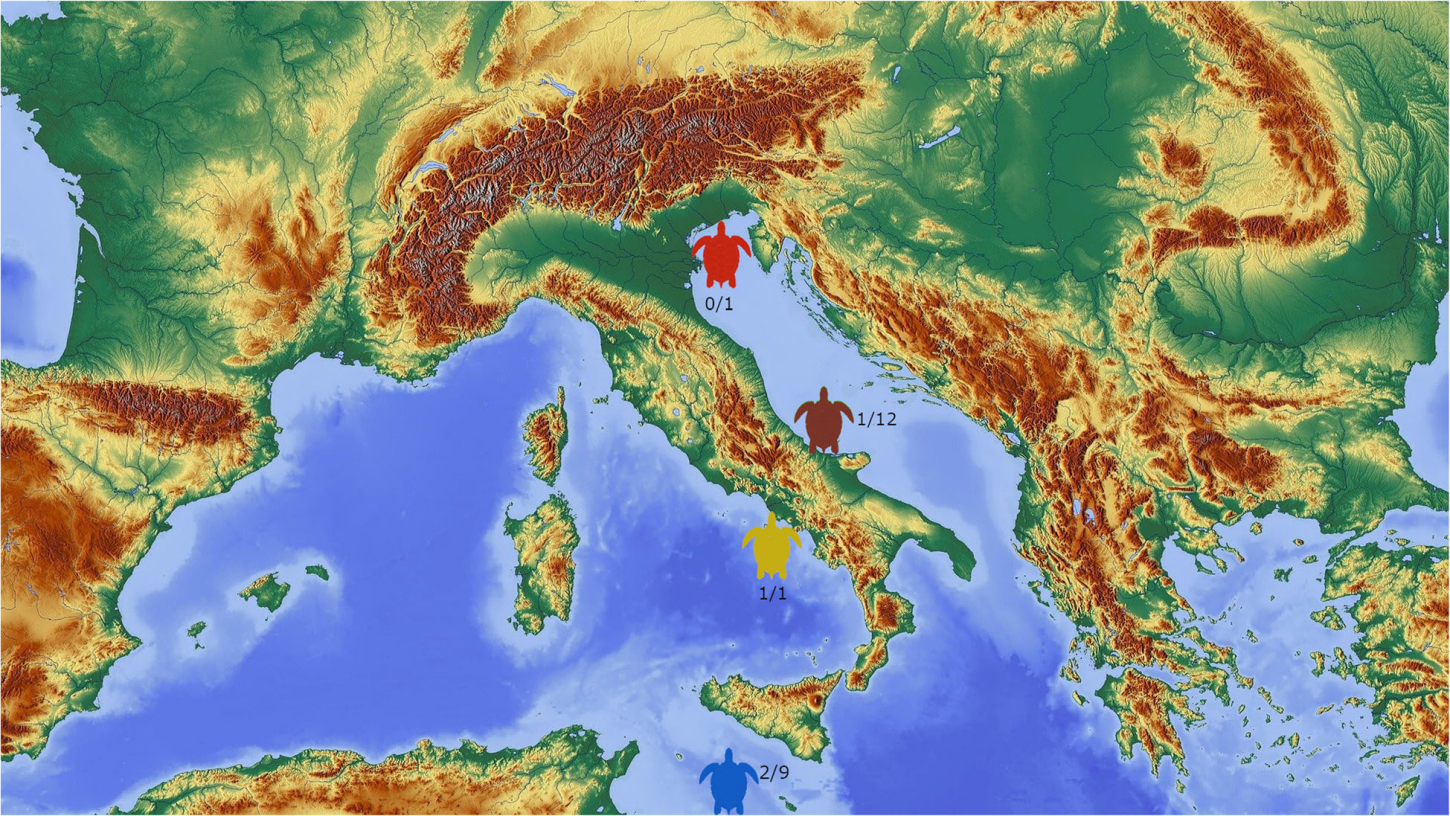
Provenence of samples included in this study. Red turtle: Centro Recupero Animali Selvatici Il Benvenuto (Rovigo, Veneto); brown, CRTM Pescara (Abruzzo); blue, CRT of Lampedusa (Agrigento, Sicily); yellow, Stazione Zoologica Anton Dohrn (Naples, Campania) Number of positive loggerhead turtles either by copromicroscopy or real time assay on blood is reported over the total number of samples collected from each locality. (map source: pixabay.com)

Three out of 23 (13.0%) loggerhead turtles were positive to *Hapalotrema*-like eggs by coprological examination. Molecular analyses allowed identification of the eggs to the species *H*. *mistroides* (accession number: LT617052.1) in all cases. Positive samples were detected from all investigated regions including one each from CRTM “L. Cagnolaro”, Stazione Zoologica Anton Dohrn, and from CRT of Lampedusa (Fig. 1), representing the eastern, western and central Mediterranean Sea respectively.

### Real time PCR on blood products

BLAST search results showed that primers and probe used in this study matched specifically to the sequences of *H. mistroides* and did not match with those of *Neospirorchis* sp. and bacteria tested. Fluorescent signal for the TaqMan real time PCR was generated when *H*. *mistroides* control DNA was tested, whereas no signal was registered when negative controls were used as templates. Limit of detection was 0.6 eggs (< 10 pg/μl, instrumental limit of fluorometer), with CT values of 39.26. The slope of the standard curve (− 3.35) was used to calculate the efficiency value (92%) and correlation coefficient (0.99).

Three out of 23 blood samples were positive by Taqman real time PCR with CT values ranging from 31.36 to 39.26, corresponding to a DNA quantity of < 10 pg. The positive results of this assay were associated in two cases with positive coprological examination, while in one case only blood tested positive, with negative result at copromicroscopy. On the counterside, one turtle which had tested positive at the coprological examination proved negative at the real time assay on blood.

Sequencing of eggs extracted from the spleen of other, previously necropsied, infected turtles and used as positive controls, confirmed them to be *H. mistroides* (Marchiori et al. 2017).

## Discussion

The presently developed PCR assay represents the first successful detection of spirorchiid DNA within blood samples of turtles. Similarly, DNA of *Schistosoma haematobium* and *Schistosoma mansoni* (Schistosomatoidea: Schistosomatidae) has been detected in blood samples from infected human patients [11, 12]. A Taq-man real time PCR was also developed to detect DNA of *Cardicola* spp. (Schistosomatoidea: Aporocotylidae) in blood samples of Bluefin Tuna [13], demonstrating higher sensitivity than traditional methods in the diagnosis and being the first non-lethal diagnostic method in this host species. Substantial discrepancies exist concerning phenotype, site of infection and life cycle of parasites of the three families of the Schistosomatoidea; nevertheless, we can speculate that cardiovascular flukes release parasitic DNA within the bloodstream of the host through similar mechanisms, regardless of parasite and host species. Cell-free DNA is considered responsible for positive results of PCR in both human and fish hosts [11, 13], resulting from high cellular turn-over of the tegument of maturing schistosomules in acute infections, as well as from degrading specimens or from circulating eggs during the chronic phase [12, 13]. Similarly, positive blood samples from this study were microscopically observed to verify the presence of intact or broken circulating eggs before extraction with no positive results, so that in these turtles the presence of cell-free DNA within the bloodstream is likely. As seen through the quantification of DNA by the present real time assay, the amount of genomic material of *H*. *mistroides* found in the blood of the sampled loggerhead turtles is very low (less than 10 pg in the whole volume tested). An increase in the volume of analysed blood could reduce the probability of false negative results: 2 ml serum samples indeed were the optimal volume for the detection of DNA of *Schistosoma* spp. in humans [12]. Nevertheless, limitations to the volume of blood drawn must be taken into account when dealing with small turtles or with animals undergoing rehabilitation following severe clinical conditions. We can suspect that the false negative result in one of the turtles here screened can be likely related to a lower amount of circulating DNA, which was out of the sensitivity limits of the developed test. In this case, indeed, the volume of blood tested was lower than the preferred amount of 400 μl, due to low volume of blood drawn from this turtle. Moreover, increasing the number of cases is surely necessary to analyse which factors can affect the amount of circulating DNA of spirorchiid parasites in infected loggerhead turtles, including the moment of infection (acute vs chronic) or parasitic burden. Collection of clinical data, eventually associated to post-mortem findings, will be thus of help in the future to clear these issues. Nevertheless, the assay was able to reveal the presence of *H*. *mistroides* infection within the blood of one loggerhead turtle which had been classified as negative by copromicroscopy. This finding highlights the potential utility of blood examination for a prompt diagnosis in hospitalized sea turtles.

Future studies should be directed toward the development of other species or genus-specific probes, in particular for those species which are less likely to shed their eggs with the feces, like neurotropic genotypes of *Neospirorchis* sp. [2, 14] Finally, testing live individuals in the Mediterranean Sea could help increasing the still scarce knowledge about distribution and pathology of spirorchiidosis among the resident population of sea turtles, overcoming the bias due to sampling only dead animals.

## Conclusions

Ante-mortem rapid, highly sensitive molecular assays can represent an important tool for a rapid and accurate diagnosis and treatment of spirorchiidosis and may be useful for surveillance programs in wild animal populations. This study represents the first evidence of the presence of circulating DNA of Spirorchiidae within the bloodstream of infected live sea turtles. New perspectives are open for the diagnosis of infection by other spirorchiid species which do not shed eggs with feces, including the highly pathogenic genotypes of *Neospirorchis*.

## Methods

### Sample collection

Between January and September 2019, 23 fecal and blood samples were collected from loggerhead turtles during convalescence period in Italian rehabilitation facilities, namely Centro Recupero Animali Selvatici Il Benvenuto (RO)(*n* = 1) and Centro Recupero Tartarughe Marine (CRTM) “L. Cagnolaro” Centro Studi Cetacei Onlus (PE) (*n* = 12) for the Adriatic coastline; Centro Recupero Tartarughe Marine (CRT) of Lampedusa (AG) (*n* = 9) for Central Mediterranean and Stazione Zoologica Anton Dohrn (NA) (*n* = 1) for the Tyrrhenian coastline (Fig. 1). Blood was collected from cervical dorsal sinus of the turtles, stored in 1 ml tubes containing lithium heparin or EDTA and frozen at − 20 °C. Feces were collected from the tanks where the same turtles were individually hospitalized and the samples were stored at − 20 °C. All samples were moved to the Department of Animal Medicine, Production and Health of the University of Padova for successive analyses.

Collection of blood samples from the turtles was conducted during clinical investigations. In accordance with the National Guidelines of Italian Ministry of Health for the care and use of animals, the non-experimental clinical veterinary practice does not require permissions.

### Coprological analyses

Fecal samples underwent qualitative copromicroscopic examination by means of a common concentration-flotation technique with high s.g. solution [10]. Eggs of spirorchiids were identified following keys in literature [2].

When fecal samples were positive for *Hapalotrema*-like eggs, DNA was extracted from frozen aliquots using PSP® Spin Stool DNA Kit (Invitek GmbH, Germany) according to manufacturer’s instructions. Amplification of the internal transcribed spacer 2 (ITS2) region of the rDNA was carried out using methods described in Stacy et al. (2010) [15]. The consensus sequences obtained were compared with the non-redundant database available in GenBank using the software BLAST [16].

### Real time PCR

DNA was extracted from 400 μl whole blood with the NucleoSpin Tissue® kit (Macherey-Nagel) following manufacturer’s instructions and stored at − 20 °C.

Specific primers and probe were designed based on *H*. *mistroides* ITS2 fragment sequence (accession number: LT617052.1) from GenBank. Primer forward (5′-ACGACGCACATTTAGTCGTG-3′), reverse (5′-AAATATGCCGCACAATAGGC-3′) and fluorescent-labeled hydrolysis probe (5′-TCCTAATTTTTCCGGTGCAG-3′) were developed. For real time monitoring of the PCR signal the probe was labelled with a 5′-FAM reporter and a Black Hole Quencher at the 3′ end (Macrogen, Korea). The amplicon size was 291base pairs.

The 20 μl reactions, containing 2.5 μl of DNA, 1X QuantiNova pathogen Master Mix (Qiagen, Valencia, CA, USA), 0.8 μM of each primer and 0.25 μM of TaqMan probe, 1 μl of internal control (IC) and 1X QuantiNova IC Probe Assay and DNA-free water, were run in a Roche Light Cycler® 96 (Roche Diagnostics, Mannheim, Germany). Cycling comprised an initial heat activation at 95 °C for 2 min, 40 2-step cycling of denaturation at 95 °C for 5 s, and combined annealing-extension step at 60 °C for 30 s. Data acquisition were performed during the combined-extension step. Each assay was performed in duplicate.

### Confirmation of PCR specificity and sensitivity

Primer and probe design was verified with Primer3 oligo analyser software (http://bioinfo.ut.ee/primer3-0.4). Their specificity was checked in silico by BLAST Analysis. The analytical specificity of the assay was tested on a panel of control samples including positive control DNA obtained from *H*. *mistroides* eggs (contaminating both water and blood samples) -previously isolated from splenic parenchyma of loggerhead turtles and molecularly identified (details of turtles, isolation methods and sequences accession numbers are reported in [10, 17]) and negative samples including negative blood, closely-related non target cardiovascular flukes geographically overlapped to *H. mistroides* in the area (i.e. *Neospirorchis* sp.) and bacteria (Enterobacteriaceae, *Citrobacter* spp., *Vibrio* spp., *Photobacterium* spp.).

Standard curves and limit of detection of the assay (LOD) were determined by using 10 2-fold dilution series of DNA from sterile water previously additioned with 100 eggs of *H*. *mistroides*. Each dilution was quantified by fluorometry (Qubit dsDNA HS assay Kit, ThermoFisher Scientific) with a limit of quantification of 10 pg/μl. The highest dilution with a positive signal in duplicate indicated the LOD.

The specificity of the assay was confirmed by end point PCR and sequencing of the PCR products. Positive controls were amplified using Platinum Taq DNA polymerase kit (Invitrogen) as described by the producer with the same couple of primers used in the real time PCR assay. Both strands of each amplicon were sequenced (Macrogen, Korea) and then compared with those present in GenBank (LT617052.1).

## Data Availability

The datasets used and analysed during the current study are available from the corresponding author on reasonable request.

## Abbreviations

CRT: Centro Recupero Tartarughe
CRTM: Centro Recupero Tartarughe Marine
LOD: Limit of Detection
